# Overcoming Barriers of Age to Enhance Efficacy of Cancer Immunotherapy: The Clout of the Extracellular Matrix

**DOI:** 10.3389/fcell.2018.00019

**Published:** 2018-03-01

**Authors:** Mark Owyong, Gizem Efe, Michael Owyong, Aamna J. Abbasi, Vaishnavi Sitarama, Vicki Plaks

**Affiliations:** ^1^Department of Anatomy, University of California, San Francisco, San Francisco, CA, United States; ^2^University of Miami Miller School of Medicine, Miami, FL, United States; ^3^Department of Orofacial Sciences, University of California, San Francisco, San Francisco, CA, United States

**Keywords:** aging, extracellular matrix, cancer immunotherapy, immunosenescence, tumor microenvironment, elderly

## Abstract

There is a growing list of cancer immunotherapeutics approved for use in a population with an increasing number of aged individuals. Cancer immunotherapy (CIT) mediates tumor destruction by activating anti-tumor immune responses that have been silenced through the oncogenic process. However, in an aging individual, immune deregulation is positively correlated with age. In this context, it is vital to examine the age-related changes in the tumor microenvironment (TME) and specifically, those directly affecting critical players to ensure CIT efficacy. Effector T cells, regulatory T cells, myeloid-derived suppressor cells, tumor-associated macrophages, and tumor-associated neutrophils play important roles in promoting or inhibiting the inflammatory response, while cancer-associated fibroblasts are key mediators of the extracellular matrix (ECM). Immune checkpoint inhibitors function optimally in inflamed tumors heavily invaded by CD4 and CD8 T cells. However, immunosenescence curtails the effector T cell response within the TME and causes ECM deregulation, creating a biophysical barrier impeding both effective drug delivery and pro-inflammatory responses. The ability of the chimeric antigen receptor T (CAR-T) cell to artificially induce an adaptive immune response can be modified to degrade essential components of the ECM and alleviate the age-related changes to the TME. This review will focus on the age-related alterations in ECM and immune-stroma interactions within the TME. We will discuss strategies to overcome the barriers of immunosenescence and matrix deregulation to ameliorate the efficacy of CIT in aged subjects.

## Introduction

### Statistics showing age-related increase in incidence of cancer

**Figure 1:**
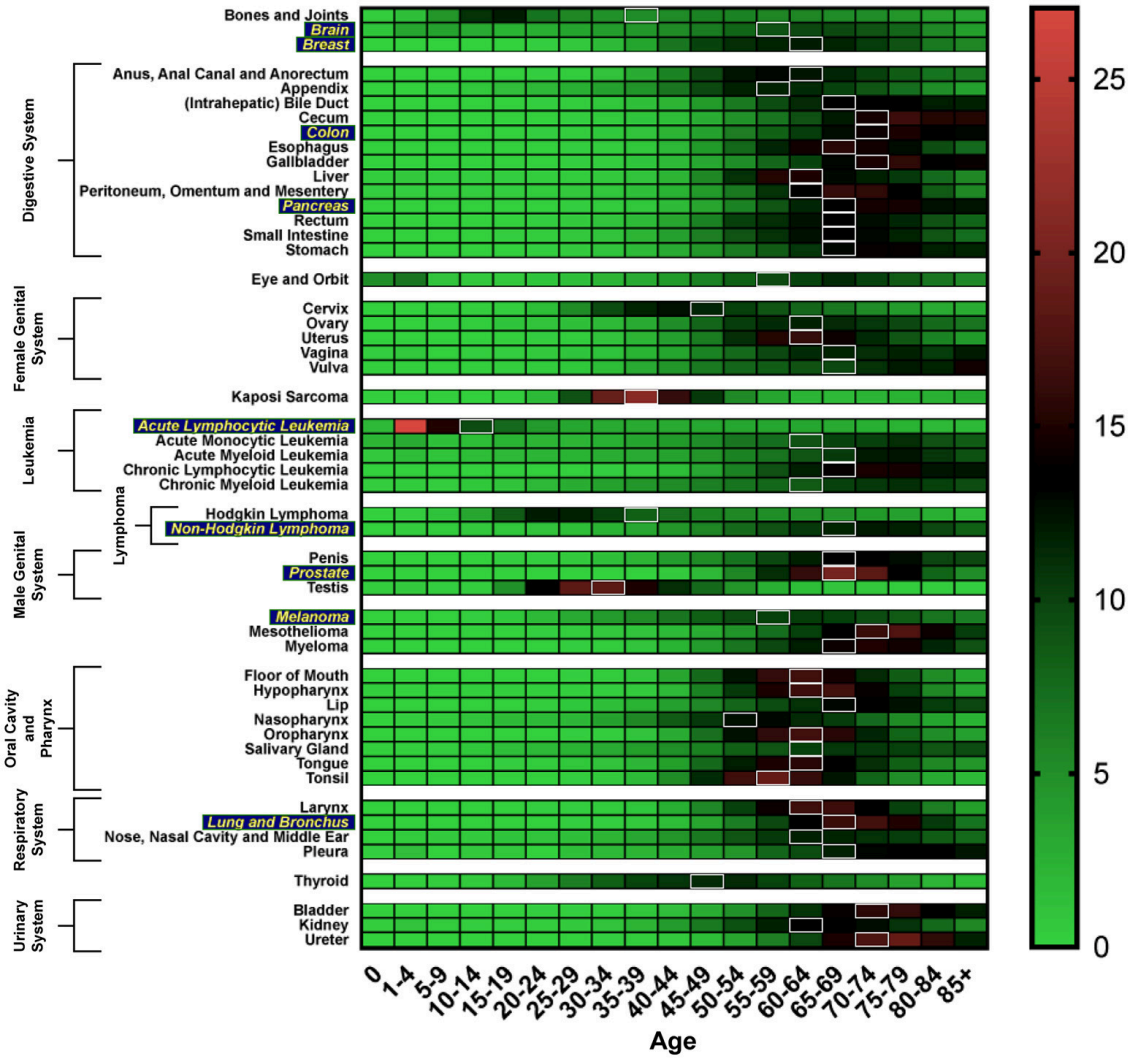
Heat map depicting incidence of cancer diagnosis by age group, analyzed using the SEER database (Howlader et al., 2017). White boxes represent the median age range of cancer incidence. Cancer types more susceptible to the aging-related impact on efficacy of CIT are in yellow.

Cancer can be considered an age-related disease. In general, the incidence of cancer increases with age, up until the age of 75, with a 39% lifetime risk of being diagnosed with any type of cancer. The median age of any cancer diagnosis is 66 years (Howlader et al., 2017). Figure 1 shows the age distribution of incidence of cancer diagnosis across various cancers. Moreover, the elderly population in the United States is expected to grow to such a degree that by 2050, the estimated population aged 65 and over will double its size as compared to that in 2012 (White et al., 2014).

### Age biases in cancer immunotherapy clinical trials

Older patients are substantially underrepresented in cancer treatment studies, in particular cancer immunotherapy (CIT) clinical trials. The Southwest Oncology Group conducted a study to analyze the data on patient enrollment in clinical trials from 1993 to 1996 (Hutchins et al., 1999). They found that patients aged 65 or older made up only 25% of the enrolled population compared to 63% in the respective United States cancer population. This underrepresentation of elderly individuals was most notable in breast cancer where only 9% of elderly women, out of the 49% in the United States breast cancer patient population, were enrolled into clinical trials. Similar trends have subsequently been found in studies analyzing trials sponsored by the National Cancer Institute both here in the United States (Lewis et al., 2003) and in Canada (Yee et al., 2003), as well as in drug registration trials with the United States Food and Drug Administration (Talarico et al., 2004).

In this mini-review we provide a systematic overview of key age-related immune alternations that cause landscape remodeling of the tumor microenvironment (TME). Adding to other recent reviews detailing the impact of aging on CIT (Hurez et al., 2016; Daste et al., 2017; Elias et al., 2017; Yousefi et al., 2017), here we focus on how age-related changes to the extracellular matrix (ECM) affect immune and other stromal cells that are the chief targets for CIT.

## Effects of age on the tumor microenvironment

### Extracellular matrix

Studies to date have largely overlooked the age-related changes to the ECM with respect to CIT efficacy (Quail and Joyce, 2013). Changes in cytokines, laminins, and collagens that alter the surrounding matrix (Sprenger et al., 2010), mitogens, and/or key enzymes [i.e., matrix metalloproteases (MMPs) and tissue inhibitors of metalloproteinase (TIMPs)] (Ruhland et al., 2016), all contribute to senescent cells creating a local inflammatory phenotype more permissive to tumor growth. The angiogenic stimulus acts as an inflammatory activator, causing inflammatory cells to produce growth factors, cytokines, chemokines, and MMPs contributing to ECM degradation or partial modification of ECM molecules that are all promoting tumor growth (Neve et al., 2014; Mongiat et al., 2016). Activation of the inflammasome (i.e., IL-1, IL-18, and NKκB) from mechanical stress on the aging ECM results in inflammation and immunodeficiency in aged subjects (Moreau et al., 2017). As a result, T cell mobility and apoptosis in elderly individuals is decreased. Stromal-derived factors also influence the ECM's pro-tumorigenic TME, which have been implicated in the regulation of senescence and malignant transformation (Acosta et al., 2008; Kuilman et al., 2008; Tchkonia et al., 2013).

Photoaging-related changes in collagen are associated with a reduction of fibrillar collagen mediated by secreted proteases, most notably MMP12 (Pittayapruek et al., 2016; Freitas-Rodríguez et al., 2017). Decreased collagen I levels and altered levels of laminins in the elderly negatively affect resident and infiltrating immune cells (Sprenger et al., 2010). On the other hand, MMPs' degradative potential in tumorigenesis tend to be dependent on the tumor type, rather than on age (Reed et al., 2000; Parikh et al., 2017). The lack of a specific and pervasive age-related effect on MMP regulation is of importance because the ability of solid tumors to express gelatinases (MMP2/9) is correlated with adverse pathological characteristics and clinical outcomes (Mancini and Di Battista, 2006). Studies on prostate tumors in aged mice further support the role of collagen I and gelatinase in vascular in-growth and tumor progression (Reed et al., 2007). It can be postulated that modification of the TME by modulating the ECM can reduce a blood vessel's efficiency; thus, impinging on drug delivery and efficacy. Taken together, ECM regulation is a therapeutic niche that can be exploited to decrease angiogenesis and subsequent tumor progression. Moreover, this and the upcoming sections below highlight the relevance of targeting MMPs and other ECM degrading proteins to specifically alter the immune landscape within the TME through ECM modulation to enhance CIT efficacy (Pickup et al., 2014; Bhome et al., 2016). This may have a direct impact on the aged TME.

### The predominant immune and stromal cells within the TME relevant to CIT

#### Cancer associated fibroblasts

Fibroblasts are a stromal cell population within the ECM; activated fibroblasts are known as cancer associated fibroblasts (CAFs) due to their role in maintaining a permissive TME for cancer cell survival and proliferation (Tao et al., 2017). With an accumulation of senescent fibroblasts during aging, it has been shown that senescent fibroblasts co-cultured with pre-malignant epithelial cells caused a marked increase in proliferation and tumorigenicity *in-vitro* and *in-vivo* (Lawrenson et al., 2010). Senescent CAFs from aged humans produce growth-promoting chemokines, such as Ccl-5, that also cause enhanced angiogenesis (Eyman et al., 2009). In addition, breast cancer cell lines mixed with fibroblasts in a xenograft model demonstrated an increase in tumorigenicity mediated by MMPs (Lawrenson et al., 2010). Therefore, CAF accumulation enables a more permissive oncogenic TME to contribute to the rise of cancer incidence in the elderly and specifically impacts the immune landscape, as discussed below.

#### Effector T cells—T helper cells and cytotoxic T cells

CIT has predominantly been focused on targeting effector T cells. However, the condensed ECM in elderly individuals serves as a biophysical barrier preventing T cells from invading the tumor and localizing around target tumor cells (Bhome et al., 2016). In aged mice, cytotoxic CD8 T cells have been shown to decrease in number with age (Lustgarten et al., 2004). The expansion of CD8 T cells is also impacted by the reduced expression of CD40 in older individuals (Elias et al., 2017). In addition, naïve CD4 T cells in aged mice proliferate less, produce significantly reduced levels of IL-2, and show poorer differentiation than those cells from young mice (Lustgarten et al., 2004). For effector T cells and natural killer (NK) cells to take action against solid tumors, they must leave the vasculature, enter the interstitium, and infiltrate the tumor mass. However, along the way they face many obstacles, most notably the impediment of the ECM (Edsparr et al., 2011). Degradation of the ECM adjacent to the tumor islets dictates T cell migration behavior and changes in T cell morphology, restricting its access to cancer cells (Salmon, 2012). Typically, TNFα and IL-6 bear anti-inflammatory roles in the late stages of disease and help mitigate the loss of function in CD4 T cells (Hurez et al., 2016), while PD-1/L1 increases proliferation of CD8 T cells (Francisco et al., 2009). With the role of the ECM as a barrier against effector T cells, immunosenescence plays a vital role in curtailing the effector T cell response within the TME and promotes a more permissive immunosuppressive microenvironment for tumorigenesis in elderly hosts.

#### Regulatory T cells

Regulatory T cells (Tregs), a subset of CD4 T cells characterized by FoxP3 and CD25 expression, suppress the anti-tumor immune response and restricts the expansion and differentiation of effector T cells (Ha, 2009). The contribution of Tregs to the age-associated decline in immune response is widely contested by some studies. In general, an increase in Tregs is associated with a worse prognosis for cancer patients, most notably those with metastatic melanoma (Ha, 2009).

In mice, an age-related increase in Treg number and expression contributed to greater immune suppression compared to Tregs from young mice (Garg et al., 2014). Moreover, immune deficiency was noted in old vs. young mice due to elevated levels of effector Tregs; systemic depletion of Tregs may concurrently elicit deleterious autoimmunity (Tanaka and Sakaguchi, 2017). When using anti-CD25 to deplete Tregs, the elevation of IFNγ and IL-17 in aged mice restored the primary and memory anti-tumor T cell responses (Sharma et al., 2006; Hurez et al., 2017). Although Tregs from young and elderly individuals inhibited T cell proliferation similarly, the levels of IL-10 were lower in aged Tregs (Fessler et al., 2013). As our understanding of CIT evolves, more studies are required in elderly subjects to support Treg mediation for attenuating tumor immune dysfunction and its subsequent effects on the ECM.

#### Myeloid-derived suppressor cells

Myeloid-derived suppressor cells (MDSCs) are a heterogeneous population of activated immature myeloid cells (Kusmartsev and Gabrilovich, 2006). During cancer, inflammation, and infection, the MDSC population expands and contributes to the negative regulation of immune responses (Gabrilovich and Nagaraj, 2009). Interestingly, Treg depletion increased MDSCs, but Treg depletion with MDSC depletion restored anti-tumor immunity and alleviated immune suppression in tumor-bearing aged mice (Hurez et al., 2012). This resulted in slowing tumor growth similar to only deleting Tregs in young hosts. Co-depletion of Tregs and MDSCs elevated cytotoxic CD8 T cells and IFNγ-producing CD4 and CD8 T cells in aged mice (Hurez et al., 2017). This potentially functions to modulate the TME's alterations of ECM components that affect signaling pathways for Arg and Nos levels. To our knowledge, co-depletion is the only immunotherapeutic approach that is primarily effective in aged, not young, hosts. Although the age effects of MDSCs on CIT in humans have not yet been reported, MDSCs are an attractive target to mitigate cancer-associated immune dysfunction in aged hosts.

#### Tumor-associated macrophages

Tumor-associated macrophages (TAMs) support tumorigenesis by promoting invasion and metastasis, tumor cell proliferation, and angiogenesis (Liu and Cao, 2014), while reducing the cytotoxicity and viability of T cells and NK cells (Edsparr et al., 2011). The pro-inflammatory (anti-tumorigenic) TAM, classically known as M1 polarized, develops in response to elevated levels of IFNγ/TNFα; the anti-inflammatory (pro-tumorigenic) TAM, classically known as M2 polarized, develops in response to elevated levels of IL-4/TGFβ (Owyong et al., 2017). Cytokines, produced through an inflammatory response, are used as predictive biomarkers for tumor development or regression (Martins et al., 2016). Interestingly, bone marrow (BM) derived and splenic TAMs, specifically M2 macrophages, increase in elderly mice and are hyper-responsive to tumor-derived factors (Jackaman et al., 2017). This was further validated in elderly patients, in which immunosuppressive M2 macrophages are elevated in lung, muscle, and lymphoid tissues (Jackaman et al., 2017). Consequentially, the age-related decrease in macrophages results in a reduction of major histocompatibility class II (MHCII) for antigen recognition alongside a reduction in IL-12 (Daste et al., 2017). We discuss the impact of decreased MHCII antigen presentation on CIT later in this mini-review.

Mice bearing different stages of Dalton's lymphoma showed that TAMs in old mice are inhibited with respect to cell binding cytotoxicity and expression of inducible nitric oxide synthase, a functional marker for M1 macrophages (Khare et al., 1999). Recent studies have illuminated a therapeutic potential utilizing anti-IL-10 to target tumor-associated M2 macrophages for a phenotypic change to M1 macrophages in young colon cancer-bearing mice (Guiducci et al., 2005). This was supported by another approach targeting TAMs with IL-12 to reprogram TAMs *in situ* within a young-aged model system (Watkins et al., 2007). However, whether this approach will be an effective treatment option in elderly individuals still requires further validation.

#### Tumor-associated neutrophils

Tumor-associated neutrophils (TANs) are phenotypically distinct from circulating neutrophils in their cytokine and chemokine profiles (Hurt et al., 2017). TANs are not well characterized throughout human tumor development or in young vs. elderly hosts.

Although studies of TANs are currently limited to young murine models, they have been shown to exhibit functional roles in either supporting cancer initiation through angiogenesis and metastasis or restricting cancer progression through expression of anti-tumor and cytotoxic mediators (Mantovani et al., 2011; Sionov et al., 2015). The most notable murine study examining TAN polarization (i.e., N1 vs. N2) indicates that anti-TGFβ can augment CIT by promoting a pro-inflammatory N1 polarization (similar to M1 macrophages) and subsequently promote the recruitment and activation of intra-tumoral CD8 T cells (Fridlender et al., 2009). In a pancreatic cancer mouse model, TANs were potent promoters of tumor angiogenesis through elevated expression of MMP9 (Nozawa et al., 2006). This was also seen in human hepatocellular carcinoma where there was a correlation between MMP9, neutrophils, and angiogenesis (Kuang et al., 2011). While it has been shown that TANs can be recruited to the tumor from splenic or BM-derived pools, further studies are required to determine if the elderly host's TME promotes an immunosuppressive N2 polarization (Cortez-Retamozo et al., 2012; Jackaman et al., 2017). With aging-associated deregulation of MMPs, N2-polarized TANs can enhance tumorigenesis in response to elevated levels of MMPs. These studies also reiterate the importance of targeting the ECM through MMP inhibition, gelatinases in particular, to specifically cause a differential immune response/recruitment to the TME.

## Control of cancer progression through CIT

**Figure 2:**
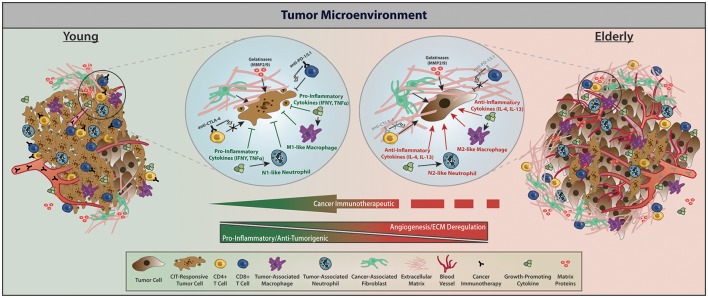
With respect to factors affecting CIT, the TME of young vs. elderly hosts differs predominately in immune infiltration and cytokine profile, regulation of angiogenesis and the ECM, as well as interactions between tumor and stromal cells. Within the young TME, increased presence of CIT-responsive tumor cells, less angiogenesis and ECM deregulation, elevated immune infiltration, and elevated pro-inflammatory cytokines give rise to a relatively more CIT-responsive TME, subsequently contributing to apoptotic tumor cells with reduced proliferation. Within elderly TMEs, relatively bigger tumors but with fewer CIT-responsive tumor cells, more angiogenesis and ECM deregulation, decreased immune infiltration, and elevated anti-inflammatory cytokines contribute to reduced apoptosis and increased proliferation subsequently enabling tumor growth. Treatment with CIT in elderly individuals triggers a phenotypic landscape remodeling toward a TME with young characteristics through enhancing the function of effector T cells following CIT and promoting a pro-inflammatory TME. Within elderly hosts, the tumor stroma permits a deregulated ECM that creates a biophysical barrier preventing effective function of effector T cells, but CIT combinations may help alleviate these age-related TME dysfunctions and decrease tumor burden. Of note—while we acknowledge that there are dormant and/or tumor initiating cells, we only depict CIT responders vs. non-responders within the TME (Gonzalez et al., 2017).

Advances in CIT have taken groundbreaking strides in treating primary and metastatic cancers. Of specific interest are the checkpoint inhibitors, which function by removing the “brakes” on the immune system, subsequently modulating the amplitude of immune responses (Yousefi et al., 2017). Another key aspect of CIT, chimeric antigen receptor T (CAR-T) cell therapy, uses an adoptive cellular therapy approach to enhance the adaptive immune response. Figure 2 provides the tumor-stromal and tumor-immune cell dynamics within the TME for young vs. elderly individuals and the interactions with targets of currently approved CIT drugs. Although other checkpoint inhibitors and immunotherapeutic approaches in the preclinical and clinical space exist, below we discuss the current molecular targets of immune checkpoints and CAR-T that have been approved by the FDA for therapeutic use.

### Current cancer immunotherapeutic molecules and target cell types

#### Checkpoint inhibitors

##### αPD-1/L1

Changes that affect the CD8 T cell compartment occur earlier than those affecting the CD4 T cell compartment. This results in only 30% of elderly patients retaining a CD8 population that is potentially efficient for CIT checkpoint inhibition (Daste et al., 2017). Programed cell death 1 (PD-1), predominantly functioning through CD8 immune modulation by binding its ligands PD-L1 (B7-H1) and PD-L2 (B7-DC), reduces T cell proliferation and promotes an evasion of the immune response (Sgambato et al., 2017). In aging mice, CD3^+^CD8^+^ T cells up-regulate the expression of PD-1 (McClanahan et al., 2015). Nivolumab, an FDA approved anti-PD-1 inhibitor, showed similar toxicity levels between younger (<65 years old) and older (65+ years old) patients (Sgambato et al., 2017). Data examining 30 patients with thymic epithelial tumors demonstrated that chemotherapy could change the TME and induce PD-L1 expression and tumor-infiltrating immune cells. Taken together, inhibiting PD-1/L1 expression increases CD8 T cell activation and potentially restores CIT efficacy from aging-related immunosenescence (Maleki Vareki et al., 2017).

##### αCTLA-4

Cytotoxic T-lymphocyte associated protein 4 (CTLA-4) delivers direct inhibitory signals to T cells, sequesters CD80 and CD86 from the surface of antigen-presenting cells, down-regulates T helper (Th) cells, and enhances Treg immunosuppressive activity (Elias et al., 2017). Additionally, the lack of CD28 expression from about 50% of the total CD4 T cell pool in adults over 65 years of age contributes to reduced T cell function in the elderly and less than optimal T cell activation (Czesnikiewicz-Guzik et al., 2008). Similarly, an increased expression of senescent CTLA-4 in the elderly contributes to inefficient activation of T cells when an antigen is presented (Daste et al., 2017).

Ipilimumab, an FDA approved anti-CTLA-4 inhibitor, has been shown to extend overall survival in elderly patients with metastatic melanoma (Chiarion Sileni et al., 2014). Although immunosenescence is a critical factor when assessing the efficacy of ipilimumab, treatment has provided a consistent survival benefit in patients (Chiarion Sileni et al., 2014). A clinical study in multiple myeloma patients showed that patients with higher expression of IFNγ, IFNγ-inducible genes, and Th1-associated markers achieved better clinical response to ipilimumab, which suggests that an activated immune microenvironment can be used as a biomarker of response (Maleki Vareki et al., 2017).

##### Combination with standard-of-care

Many studies on the effects of aging do not use the standard-of-care combination therapy that relies on radiation therapy (RT) or (neo)adjuvant chemotherapy followed by CIT. This is attributable to our incomplete knowledge of how aging affects tumor-specific immunopathology to increase therapeutic efficacy and safety.

In the limited number of studies examining the efficacy of combination CIT in aged hosts, Treg depletion combination therapy has shown the most promise. A study in aged mice bearing B16 melanoma treated with anti-PD-L1 failed (Figueiredo et al., 2016). However, anti-PD-L1 treatment efficacy was partially restored in aged mice with lymphoma when combined with anti-CTLA-4 therapy (Mirza et al., 2010; Figueiredo et al., 2016). Most multifaceted CIT studies exhibited synergistic outcomes in young mice, but ineffective outcomes in elderly hosts. For instance, reducing immune suppression, while enhancing adjuvant effects using TLR agonists in a breast cancer mouse model worked in young, but not aged hosts (Pawelec et al., 2009). In addition, IL-2 combined with CD40 agonists to augment the immune system only showed efficacy in young mice bearing metastatic renal cell carcinoma (Murphy et al., 2003). However, treatment with combinatorial therapy exhibited toxic side effects and rapid deterioration in many organs leading to multi-organ toxic syndrome (MOTS) with marked increases in pro-inflammatory cytokines (Bouchlaka and Murphy, 2013). The severe cytokine snowball was alleviated through macrophage depletion, highlighting the critical role myeloid cells play within the TME as potential targets for CIT. More specifically, TNFα inhibition with etanercept, alongside an agonist for IL-2 and CD40, played a critical role in mediating a robust anti-tumorigenic effect in aged lung carcinoma-bearing mice with a significant increase in overall survival (Bouchlaka et al., 2013).

The limited number of studies using multifaceted immunotherapy highlights the lack of understanding of aging-related immune dysfunction to promote the development of novel CIT regimens for elderly hosts. With the field of CIT ever growing, efficacious combination therapy requires further specification with definitive biomarkers to stratify aged patients based on age-specific agents, doses, and schedules, while also assessing toxicity.

#### Chimeric antigen receptor T cells

CAR-T cell immunotherapy is indicated predominately for hematological indications (Owyong et al., 2017), and relies on engineering T cells with a tumor-specific antigen receptor that interacts with tumor-associated antigens (TAAs), independent of human leukocyte antigen (HLA) expression (Yu et al., 2017). This is of clinical relevance because one of the major impediments of TCR-based immunotherapy is TCR's recognition of antigens in an HLA-dependent pathway. However, tumors tend to down-regulate HLA. Even more so, HLA-DR is down-regulated in aged individuals (Villanueva et al., 1990). This may explain the lack of clinical success and/or attempts of checkpoint inhibition in the elderly. Therefore, CAR-T cell immunotherapy could lead to new therapeutic options by bypassing an HLA-restricted pathway.

Although CAR-T cell CIT has thus far only been approved for hematological indications, pre-clinical trials for solid tumors targeting epidermal growth factor receptor (EGFR), human epidermal growth factor receptor 2 (HER2), and mesothelin (MSLN) have all shown immense promise (Yu et al., 2017). EGFR-target TAAs in glioblastoma (GBM), HER2-target TAAs in GBM and sarcoma, and MSLN-target TAAs in mesothelioma have all demonstrated an effective on-target therapeutic response with increased overall survival leading to the development of clinical trials (Yu et al., 2017). Limitations of CAR-T CIT include the lack of an ideal TAA, an inefficient delivery method for trafficking of CAR-T cells to the tumor site, and off-target toxicities associated with immunotherapy. To our knowledge, there are currently no published studies examining the effects of aged CAR-T cells and whether there is an age-related effect on the efficacy of CAR-T cells generated from elderly hosts. However, the tumor indications listed above for CAR-T CIT predominantly have a median age of incidence between 55 and 74 (Figure 1).

## Post immunotherapy changes to immune and stromal cells within the TME and its effect on the ECM

With gaps in the clinical understanding of ECM alternations and its effects on the immune landscape in elderly individuals, our current knowledge relies heavily on pre-clinical models. Previous work has shown that anti-CTLA-4 may kill effector Treg cells or attenuate their suppressive activity. By combining Treg-cell targeting (i.e., by reducing Treg cells or attenuating their suppressive activity in tumor tissues) with the activation of tumor-specific effector T cells using immune checkpoint blockade, CIT has the potential to be more effective. One strategy for evoking effective tumor immunity without autoimmunity is to specifically target terminally differentiated effector Treg cells rather than all FoxP3 T cells. Additionally, one could hypothesize that if a cancer cell can be modified to secrete less collagen, then there will be decreased support for vascular in-growth and subsequent tumor progression. A recently developed CAR-T method using both CAR and IL-12 (referred to as armored CAR-T cells) has been shown to help T cells pass the biophysical barrier (Yeku et al., 2017). These armored CAR-T cells use heparanase, specific enzymes from T cells, to improve their ability in degrading the ECM, thereby promoting effector T cell infiltration into the tumor bed (Zhang and Xu, 2017).

Contrary to young hosts, aged individuals exhibit a toxic elevation in pro-inflammatory cytokines following a multifaceted immunotherapeutic approach (Bouchlaka and Murphy, 2013). Current studies using CIT in elderly pre-clinical and clinical studies need to be aware of the low efficacy potentially attributable to over-activation of the immune system. MOTS should also be considered in individuals receiving RT or chemotherapy and whether these effects can be ameliorated with examining novel pro-inflammatory cytokines, such as TNFα and IFNγ. Strategies to reduce immune dysfunction must be tailored to account for age-related and tumor-related immune dysfunctions for optimal utility. Thus, improved and efficacious CIT for aged hosts, who are at the greatest risk for cancer, is a realistic goal that can be met with a better understanding of the specific effects of age on the stroma and tumor-related immune dysfunction. This will allow us to tailor novel therapeutic approaches in the aging population. Lastly, we advocate for the use of pre-clinical models encompassing a pertinent age spectrum to a specific disease indication; thus, meticulously discerning the therapeutic efficacy as well as pharmacodynamic and pharmacokinetic parameters of CIT in the intended patients.

## Author contributions

MaO and VP conceptualized the ideas and designed the outline of the article. MaO, GE, MiO, and VP drafted the article with insight from AA and VS. All authors have approved the final article.

### Conflict of interest statement

The authors declare that the review was conducted in the absence of any commercial or financial relationships that could be construed as a potential conflict of interest. The reviewer YY and handling Editor declared their shared affiliation.
